# The mediating effect of health-promoting behaviors on the relationship between infertility stress and fertility-related quality of life of infertile women: a cross-sectional study

**DOI:** 10.4069/whn.2025.03.24

**Published:** 2025-03-28

**Authors:** Eun Jin Kim, Ju-Hee Nho, Hye Young Kim

**Affiliations:** 1 College of Nursing, Woosuk University, Wanju, Korea; 2 College of Nursing, Research Institute of Nursing Science, Jeonbuk National University, Jeonju, Korea

**Keywords:** Health behavior, Infertility, Quality of life, Stress, Women

## Abstract

**Purpose:**

Infertility is a global health problem that affects many people of reproductive age. This study aimed to examine the mediating effects of health-promoting behaviors (HPBs) on the relationship between infertility stress and fertility-related quality of life (QoL) in infertile women.

**Methods:**

A cross-sectional survey was conducted with 138 infertile women in Korea, who were recruited from August to October 2022, from two public health centers and two infertility clinics at obstetrics and gynecology hospitals in Jeonju, Korea. The participants completed a self-reported questionnaire via an online survey. The data were analyzed using an independent t-test, one-way analysis of variance, Scheffé test, Pearson correlation coefficients, and multiple regression analysis using PROCESS macro with 95% bias-corrected bootstrap confidence interval (CI) (5,000 bootstrap resampling).

**Results:**

The mean scores for fertility-related QoL, infertility stress, and HPB were all greater than the midpoint. Fertility-related QoL was positively correlated with HPBs (r=.20, *p*=.022) and negatively correlated with infertility stress (r=–.41, *p*<.001). The total effect of infertility stress on fertility-related QoL (B=–0.34, *p*<.001) and infertility stress on HPBs were significant (B=–0.01, *p*=.024). The effects of HPBs on fertility-related QoL (B=6.54, *p*<.001) and infertility stress on fertility-related QoL (direct effect; B=–0.30, *p*<.001) were significant. After controlling for demographic covariates, HPBs partially mediated the relationship between infertility stress and fertility-related QoL in infertile women (B=–0.03; 95% CI, –0.08 to –0.00).

**Conclusion:**

To improve fertility-related QoL for infertile women, interventions to reduce infertility stress and improve HPBs should be developed and implemented.

## Introduction

Infertility is the inability to conceive following 1 year of unprotected sexual activity without contraception. It is a global health problem affecting millions of people of childbearing age. It is estimated that 48 million couples and 186 million individuals suffer from infertility worldwide, accounting for 15% of people of childbearing age [1]. The rate of infertility can be caused by inappropriate health-promoting behaviors (HPBs) caused by lifestyle changes, such as obesity, stress, unbalanced eating habits, lack of physical activity, and smoking [2-7].

Fertility-related quality of life (QoL) refers to the QoL of men and women experiencing infertility problems [7]. When women experience reproductive disorders during their reproductive years, their fertility-related QoL can be compromised [8]. As a result of the diagnosis of infertility and the procedure itself [9], infertile women have a lower fertility-related QoL and have difficulty coping with high levels of psychological distress [8]. This is because most women complete their married life by fulfilling their social identity and gender roles. As infertile women are unable to realize these social expectations, their fertility-related QoL decreases due to stress [8]. It has been found that Korean infertile women have slightly lower fertility-related QoL scores than Western infertile women in most of the areas of fertility-related QoL, including physical, emotional, social, and relational [10].

Infertility stress is characterized by identity crisis, social isolation, stigma, sexual stress, and financial strain. It leads to the discontinuation of infertility treatment, which burdens marital life [11]. Infertility stress can adversely affect patients’ psychological and emotional well-being during the treatment process for infertility [12]. In other words, infertility stress affects a woman’s fertility through the sympathetic-adrenal-medullary pathway and has a greater influence on a woman’s life [8]. Infertile women experience greater stress in terms of overall stress, social anxiety, relationship anxiety, parental role desire, and sexual anxiety due to infertility [13], indicating that infertility-related stress in infertile women negatively impacts fertility-related QoL [14,15].

HPBs are activities intended to increase the level of personal well-being and maintain or enhance the individual’s self-realization or achievement [16]. There is a significant association between HPBs and infertility [17]. Various lifestyle habits influence fertility, and the direct correlation between lifestyle habits and female reproductive health is strengthened as the age of childbearing is delayed [18]. Compared to women without infertility, infertile women have lower reproductive HPBs [19], and their HPBs are generally less reported [20]. Moreover, infertile women scored lower than men in physical activity and health responsibility among the subdomains of HPBs [21,22]. Pregnant women and office workers with better HPBs had less stress [23,24], and infertile women with better HPBs had less depression [22]. In addition, the better the HPBs of women with polycystic ovarian syndrome, the higher the QoL [25], and the better the health-related behavior of women undergoing infertility treatment, the higher the life satisfaction [26].

Regarding this, HPBs and infertility stress of infertile women are important factors in fertility-related QoL, and healthcare providers need to take an active interest in this to improve fertility-related QoL in infertile women. Most of the studies conducted so far have fragmentarily revealed the relationship between HPBs and fertility-related QoL [25], HPBs and infertility stress [8,22,23], and infertility stress and fertility-related QoL [14,15]. However, there are not many studies that have identified the degree of HPBs, infertility stress, and fertility-related QoL in infertile women and the relationship between them. In particular, there are few studies on the mediating effect of infertility stress on the relationship between HPBs and fertility-related QoL in infertile women, thus empirical evidence is needed.

Therefore, this study aimed to identify the mediating effect of HPBs on the relationship between infertility stress and fertility-related QoL in infertile women. Specifically, this study aimed to: (1) identify the general characteristics, infertility stress, HPBs, and fertility-related QoL, (2) demonstrate the correlation between infertility stress, HPBs, and fertility-related QoL, and (3) identify the mediating effect of HPBs on the relationship between infertility stress and fertility-related QoL in infertile women.

## Methods

**Ethics statement:** This study was approved by the Institutional Bioethics Committee of Jeonbuk National University (No. 2022-05-016-001). All participants voluntarily consented to participate after being informed of the purpose of the study. They were informed that the results of the questionnaire would not be used for purposes other than the research, and that they could withdraw from the study at any time.

### Study design and participants

This study used a descriptive correlational design employing a cross-sectional survey to investigate the mediating effect of HPBs on the relationship between infertility stress and fertility QoL of infertile women. This study was described in accordance with the STROBE guidelines (https://www.strobe-statement.org/index.php?id=strobe-home).

In this study, infertile women were recruited according to the following selection criteria: (i) married women aged 19 years or older, (ii) women with primary or secondary infertility who have failed to conceive despite maintaining a normal marital relationship for more than 1 year, (iii) women diagnosed with infertility by a physician, (iv) women undergoing treatment at a fertility clinic or planning to start treatment, and (v) women who understood the purpose of the study and agreed to participate.

Exclusion criteria were (i) women not living with spouses and (ii) those self-reporting serious mental illness (e.g., depression, anxiety disorders).

The target number of participants for this study was calculated using the program G*Power 3.1.9.7. Based on the effect size (ΔR^2^=.09–.33, R^2^=.22–.40) and predictors (e.g. age, duration in infertility, burdensome infertility, 10–16 predictors) confirmed in previous studies [15,27] on the QoL of infertile women. Thus, the effect size was set at .15, significance level .05, power .80, and 12 predictors (10 general characteristics, HPBs, and infertility stress) were input, resulting in a minimum sample size of 127 required for regression analysis. We recruited 148 participants, considering an attrition rate of 93.3% [28]. The final analysis included 138 participants, after excluding 10 cases of unreliable responses (response rate, 93.2%).

### Data collection

For recruitment, a poster containing information about the study, inserted as a quick response (QR) code link, was posted at the two public health centers and two infertility clinics at obstetrics and gynecology hospitals from Jeonju province in South Korea. Women who clicked the QR code could review the inclusion/exclusion criteria screening questions, and their participation in the online survey was considered as consenting to the study. The data were collected via online survey from August 11 to October 11, 2022. As compensation for participating in the study, a mobile beverage coupon (5,000 Korean won, approximately 3 US dollars) was provided.

### Measures

The use of all measurements in this research was approved by the developers and/or translators.

### Fertility-related quality of life

The Korean version [10] of Fertility-Related Quality of Life (FertiQoL) [7] was used to measure QoL in infertile populations. The 34 items consist of 24 items for core FertiQoL, eight items for treatment FertiQoL, and one item each for overall physical health and satisfaction with QoL. The core FertiQoL includes the emotional, mind-body, relational, and social domains; treatment FertiQoL includes the environment domain and treatment tolerability. Rated on a 5-point Likert scale (0 to 4), the total score is calculated by summing the average of the core QoL and treatment QoL domains, while excluding overall health status and QoL satisfaction. All areas are evaluated by converting scores to a 0 to 100 range, with a higher total score (possible range, 0–100) indicating a higher QoL associated with infertility [7]. The tool’s reliability Cronbach’s α was .92 at the time of development [7], and .92 in this study.

### Infertility stress

Infertility stress was measured using the Fertility Problem Inventory developed by Newton et al. [29] and adapted by Kim and Shin [30]. Each of the 46 items is rated on a 6-point Likert scale (1, strongly disagree to 6, strongly agree). The total score ranges from 46 to 230, with a higher score indicating a higher stress related to infertility. Cronbach’s α was .93 at the time of development [29], .92 in the study by Kim and Shin [30], and .80 in this study.

### Health-promoting behaviors

For HPBs, the Korean version [31] of Walker et al.’s [32] Health Promoting Lifestyle Profile version II was used. It includes 52 items, each rated on a 4-point Likert scale (1, not at all to 4, regularly). Higher scores (possible range, 52–208) indicate a greater degree of HPBs, and possible scores for the tool following six subdomains are as follows: health responsibility (9–36), physical activity (8–32), nutrition (9–36), spiritual growth (9–36), interpersonal relationships (9–36), and stress management (8–32) The Cronbach’s α was .92 in the study at the time of development [32], .94 (.80–.88 for subdomains) in the study by Hwang et al. [31], and .94 (.72–82 for subdomains) in this study.

### Statistical analysis

The data were analyzed using IBM SPSS ver. 25.0 (IBM Corp., Armonk, NY, USA). Descriptive statistics were used to evaluate participants’ general characteristics and variables. An independent t-test and one-way analysis of variance were used to identify differences in fertility-related QoL according to participants’ general characteristics. Pearson correlation was used to identify the relationship between fertility-related QoL, infertility stress, and HPBs. PROCESS macro ver. 3.5.3 (model 4, number of samplings; 5,000 using bootstrapping) [33] was employed to identify the mediating effect of HPBs on the relationship between infertility stress and fertility-related QoL.

## Results

### Participants’ fertility-related quality of life according to general characteristics.

The mean age of the participants was 35.9 years. The duration of infertility treatment was less than 2 years for 81 patients (58.7%). Additionally, 105 participants (76.1%) reported being burdened with infertility treatment; and among them, 48 (34.8%) responded that parents-in-law was the most common source of burden. The age (over 35 years; t=–2.68, *p*=.009), duration of infertility treatment (1–2 years; F=4.34, *p*=.015), and the person causing burden (no; t=–4.04, *p*<.001) showed a statistically significant high score on fertility QoL (Table 1).

### Level of fertility-related quality of life, infertility stress, and health-promoting behaviors

The mean scores for fertility-related QoL, infertility stress, and HBP were greater than the midpoint, i.e., 55.74±12.40, 158.80±16.91, and 144.86±22.64, respectively. All variables were normally distributed with skewness and kurtosis within absolute values of 2 (Table 2).

### Correlations between fertility-related quality of life, infertility stress, and health-promoting behaviors

There was a significant negative correlation of moderate strength between fertility-related QoL infertility stress (r=–.41, *p*<.001), and a significant weak but positive correlation between fertility-related QoL and HPBs (r=.20, *p*=.022) (Table 3).

### The mediating effect of health-promoting behavior on the relationship between infertility stress and fertility-related quality of life

**Figure 1.**
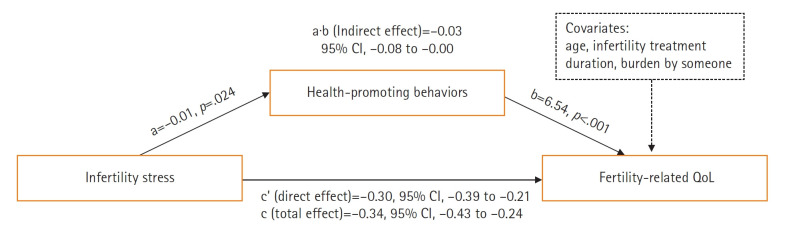
Statistical relationships for simple mediation model among fertility-related quality of life (QoL), infertility stress, and health-promoting behaviors while controlling for general characteristic variables. a: regression coefficient for infertility stress in a model predicting health-promoting behaviors by infertility stress; b and c’: regression coefficient in a model predicting fertility-related QoL by health-promoting behaviors and infertility stress; c: total effect of infertility stress on fertility-related QoL while controlling for general characteristic covariates; a∙b: indirect effect of infertility stress on fertility-related QoL mediated by health-promoting behaviors while controlling for general characteristic covariates; 95% CI: 95% bias-corrected bootstrap confidence interval.

The assumptions made in the regression analysis before examining the mediating effect of HPBs were appropriate. The Durbin-Watson index was 1.436, independent of autocorrelation. As a result of analyzing the influence using Cook’s distance statistic, there was no value showing more than 1.0 from .00 to .16, and the multicollinearity between independent variables was less than 10, with the variation inflation factor ranging from a minimum of 1.03 to a maximum of 1.11, while all the tolerance limits were above 0.1, indicating that there was no multicollinearity, which was suitable for the regression analysis. The results identify the mediating effect of HPBs on the relationship between infertility stress and fertility-related QoL (Figure 1). In the first stage of PROCESS macro analysis, the total effect of infertility stress on fertility-related QoL was significant (B=–0.34, *p*<.001). In the second stage, the effect of infertility stress on HPBs was significant (B=–0.01, *p*=.024). In the third stage, the effect of HPBs on fertility-related QoL (B=6.54, *p*<.001) and the effect of infertility stress on fertility-related QoL (direct effect, B=–0.30, *p*<.001) were significant. In the fourth stage, a significant indirect effect (B=–0.03; 95% CI, –0.08 to –0.00) of HPBs on the relationship between infertility stress and fertility-related QoL was identified (Table 4).

## Discussion

This study of 138 infertile women revealed that fertility-related QoL was positively correlated with HPBs and negatively correlated with infertility stress. Moreover, HPBs partially mediated the relationship between infertility stress and fertility-related QoL.

Our finding that infertility stress affected HPBs and fertility-related QoL is similar to prior studies that found the adverse impact of stress of the physical and psychological burden of infertility treatment on HPBs [34], with a higher stress score indicating a lower QoL [15,30,35]. Given that infertility stress is caused by physical, psychological, and social factors, efforts to improve QoL by reducing infertility stress through supportive therapy, counseling [36], coaching [37], cognitive-behavioral therapy [38], and internet-based mindfulness counseling [39,40] are needed. Relaxation therapy and nursing counseling have also been shown to be beneficial [41,42]. Healthcare providers should actively use these strategies to reduce infertility stress by engaging in prepregnancy counseling services [43] and family support, healthcare provider’s intervention, and continuous support policies for infertility treatment and procedure costs should be considered [13,42]. In addition, this is similar to previous studies that found that infertile women or reproductive women who perform healthy lifestyles such as regular physical activity or balanced diet have positive mental health outcomes (e.g., stress, depression, anxiety, and somatization) [44,45], and improvement in HPBs aids healthy pregnancy outcomes for infertile women [46].

This study confirmed that HPBs was associated with better fertility-related QoL, which is similar to previous findings that improving HPBs in infertile women improves QoL [21,47,48], that QoL improved simultaneously with better health [49], and participants who performed more healthy behaviors such as regular physical activity, ensuring a healthy diet, and maintaining sufficient rest and sleep reported higher QoL [50]. The integrational lifestyle intervention for QoL improvements requires ongoing longitudinal studies and healthcare systems to design and implement interventions.

Furthermore, given that HPBs had a partial mediating effect on the relationship between infertility stress and fertility-related QoL, stress as a risk factor and HPBs as a mediating factor should be addressed together to improve QoL in infertile women. This finding is consistent with previous studies on university students and obese women, i.e., that high perceived stress levels and depressive symptoms can negatively affect QoL [51,52]; HPBs completely mediated the relationship between perceived stress and QoL [51]; and HPBs partially mediated the relationship between social support and QoL [52]. Considering that information on HPBs has a positive effect on the fertility of infertile women [8,17], counseling and education to improve the HPBs of infertile women and raise awareness of HPBs, and focusing on physical activity and health responsibility for infertile women [21] are needed. In the future, we suggest personalized intervention research that utilizes digital healthcare to monitor the lifestyle of infertile women in real time and help them maintain a healthy lifestyle based on accumulated data.

The limitations of this study are as follows. Since this study was conducted by convenience sampling of infertile women located in one region, generalizing is limited. Second, this study confirmed subjective HPBs through a questionnaire, but it will be possible to understand it more objectively through the analysis of real-time data on lifestyle patterns. In the future, we suggest research on HPBs confirmation through wearable digital health measures. In addition, considering the wide range of infertility treatments, future research on the QoL of infertile women by specifying infertility treatment methods may offer more specific information on fertility-related QoL. Nevertheless, this study identified the mediating effect of HPBs on the relationship between infertility stress and fertility-related QoL in infertile women.

In conclusion, this study found that fertility-related QoL of infertile women had a negative correlation with infertility stress and a positive correlation with HPBs. In addition, HPBs partially mediated the relationship between infertility stress and fertility-related QoL. Therefore, it is necessary to develop interventions aimed at reducing infertility stress and increasing HPBs in order to improve the fertility-related QoL of infertile women. Nurses can use findings to develop and apply nursing interventions that can promote HPBs and reduce stress in infertile women.

## Figures and Tables

**Table 1.** Fertility-related quality of life according to participants’ characteristics (N=138)

Characteristic	Categories	n (%) or	Fertility-related QoL	
		Mean±SD	Mean±SD	t or F (*p*)
Age (year)	Range, 26–44	35.89±3.34		
	<35	43 (31.2)	51.89±10.68	–2.68 (.009)
	≥35	95 (68.8)	57.49±12.78	
Marital duration (month)	<36	35 (25.4)	58.13±13.17	1.32 (.190)
	≥36	103 (74.6)	54.94±12.09	
Religion	Yes	41 (29.7)	51.96±10.21	–1.73 (.086)
	No	97 (70.3)	56.92±13.09	
Occupation	Yes	118 (85.5)	56.59±11.90	1.97 (.051)
	No	20 (14.5)	50.76±14.38	
Residence	Rural	102 (73.9)	54.30±10.55	–1.92 (.061)
	Urban	36 (26.1)	59.82±16.06	
Monthly house income (million KRW)	2–3	9 (6.5)	55.90±20.69	0.00 (.999)
	3–5	34 (24.6)	55.79±11.78	
	≥5	95 (68.8)	55.71±11.79	
Infertility treatment	≥1, <2^a^	81 (58.7)	58.26±12.62	4.34 (.015)
Duration (year)	≥2, <3^b^	23 (16.7)	53.03±9.79	a>b, c^†^
	≥3^c^	34 (24.6)	51.58±12.26	
Burden of infertility	Yes	105 (76.1)	53.48±11.32	–4.04 (<.001)
	No	33 (23.9)	62.96±13.10	
Person causing burden^§^	Husband	5 (3.6)	61.35±13.49	1.10 (.355)
	Parents-in-law	48 (34.8)	53.74±10.76	
	Parents	6 (4.3)	55.30±4.99	
	Myself	46 (33.3)	52.11±12.12	

KRW; Korean won (one million KRW is roughly 800 US dollars); QoL: quality of life.

† Analyzed by Scheffé test.

‡ Among participants who reported experiencing burden (n=105).

**Table 2.** Fertility-related QoL, infertility stress, and HPBs in infertile women (N=138)

Variable	Categories	Possible range	Mean±SD	Minimum	Maximum	Skewness	Kurtosis
Fertility-related QoL	Overall physical health	0–4	2.33±0.76	0.00	4.00	–0.32	–0.14
	Quality of life satisfaction	0–4	2.33±0.85	0.00	4.00	–0.03	–0.18
	Total	0–100	55.74±12.40	8.33	93.23	0.00	1.15
	Core fertility-related QoL	0–100	57.71±15.06	14.58	93.75	0.36	–0.09
	Emotional subscale	0–100	57.70±18.50	0.00	100.00	0.02	–0.08
	Mind-body subscale	0–100	56.37±19.22	0.00	100.00	–0.01	–0.12
	Relational subscale	0–100	59.90±16.69	20.83	95.83	0.28	–0.59
	Social subscale	0–100	56.88±16.18	12.50	91.67	–0.03	–0.18
	Treatment fertility-related QoL	0–100	53.77±12.77	2.08	92.71	–0.30	1.26
	Environment subscale	0–100	54.38±12.52	4.17	91.67	–0.11	1.57
	Tolerability subscale	0–100	53.17±18.65	0.00	93.75	–0.24	–0.28
Infertility stress		46–230	158.80±16.91	116.00	206.00	0.21	0.40
HPB	Total	52–208	144.86±22.64	88.00	200.00	–0.01	–0.19
	Health responsibility	9–36	24.59±5.00	12.00	36.00	–0.23	–0.22
	Physical activity	8–32	21.73±4.80	9.00	32.00	–0.08	–0.30
	Nutrition	9–36	25.14±4.34	15.00	35.00	0.12	–0.38
	Spiritual growth	9–36	24.33±4.56	10.00	36.00	–0.30	0.25
	Interpersonal relationships	9–36	26.55±4.58	10.00	36.00	–0.25	0.40
	Stress management	8–32	22.51±4.42	10.00	32.00	–0.09	–0.27

HPB: Health-promoting behavior; QoL: quality of life.

**Table 3.** Correlations among fertility-related QoL, infertility stress, and health-promoting behaviors (N=138)

Variable	r (*p*)	
	Fertility-related QoL	Infertility stress
Fertility-related QoL	1	
Infertility stress	–.41 (<.001)	1
Health-promoting behaviors	.20 (.022)	–.02 (.778)

QoL: Quality of life.

**Table 4.** Mediating effect of health-promoting behaviors between infertility stress and fertility-related quality of life by bootstrapping (N=138)

Effect	Variable	B	SE	t	*p*	95% CI	P_M_
Direct	Infertility stress → Fertility-related QoL^c’^	–0.30	0.05	–6.70	<.001	–0.39 to –0.21	.088
Indirect	Infertility stress → HPBs^a^	–0.01	0.00	–2.28	.024	–0.01 to –0.00	
Indirect	HPBs → Fertility-related QoL^b^	6.54	1.73	3.78	<.001	3.12 to 9.96	
Indirect	Infertility stress → HPBs → fertility-related QoL^ab^	–0.03	0.02			–0.08 to –0.00	
Total	c’+ ab	–0.34	0.05	–7.22	<.001	–0.43 to –0.24	

CI: Confidence interval; HPB: health-promoting behavior; QoL: quality of life; P_M_=proportion mediated, ratio of the indirect effect to the total effect. Covariate: age, infertility treatment duration, and burden by someone. References: age ≥35 years, treatment duration ≥3 years, and burden by someone.

## References

[1] World Health Organization (2021). WHO fact sheet on infertility. Glob Reprod Health.

[2] Collée J, Mawet M, Tebache L, Nisolle M, Brichant G (2021). Polycystic ovarian syndrome and infertility: overview and insights of the putative treatments. Gynecol Endocrinol.

[3] Hayden RP, Flannigan R, Schlegel PN (2018). The role of lifestyle in male infertility: diet, physical activity, and body habitus. Curr Urol Rep.

[4] Skoracka K, Ratajczak AE, Rychter AM, Dobrowolska A, Krela-Kaźmierczak I (2021). Female fertility and the nutritional approach: the most essential aspects. Adv Nutr.

[5] Sun H, Gong TT, Jiang YT, Zhang S, Zhao YH, Wu QJ (2019). Global, regional, and national prevalence and disability-adjusted life-years for infertility in 195 countries and territories, 1990-2017: results from a global burden of disease study, 2017. Aging (Albany NY).

[6] Yang SR, Yeo JH (2017). Effects of irrational parenthood cognition, post traumatic stress disorder and spousal support on quality of life of infertile women. Korean J Women Health Nurs.

[7] Boivin J, Takefman J, Braverman A (2011). The Fertility Quality of Life (FertiQoL) tool: development and general psychometric properties. Fertil Steril.

[8] Palomba S, Daolio J, Romeo S, Battaglia FA, Marci R, La Sala GB (2018). Lifestyle and fertility: the influence of stress and quality of life on female fertility. Reprod Biol Endocrinol.

[9] Jung YJ, Kim HY (2017). Factors influencing infertility-related quality of life in women undergoing assisted reproductive techniques: focusing on depression and resilience. Korean J Women Health Nurs.

[10] Chi HJ, Park IH, Sun HG, Kim JW, Lee KH (2016). Psychological distress and fertility quality of life (FertiQoL) in infertile Korean women: The first validation study of Korean FertiQoL. Clin Exp Reprod Med.

[11] Woods BM, Patrician PA, Fazeli PL, Ladores S (2022). Infertility-related stress: a concept analysis. Nurs Forum.

[12] Kim M (2014). Stress, depression, and fetal attachment in pregnant women having infertility treatments. Korean J Women Health Nurs.

[13] Lei A, You H, Luo B, Ren J (2021). The associations between infertility-related stress, family adaptability and family cohesion in infertile couples. Sci Rep.

[14] Luk BH, Loke AY (2015). The impact of infertility on the psychological well-being, marital relationships, sexual relationships, and quality of life of couples: a systematic review. J Sex Marital Ther.

[15] Lee YH, Park JS (2019). Factors affecting the infertility-related quality of life among the infertility women. J Korean Soc Matern Child Health.

[16] Pender NJ (1987). Health promotion in nursing practice.

[17] Kim EJ, Nho JH (2022). Lifestyle interventions for adults with infertility. J Lifestyle Med.

[18] Bala R, Singh V, Rajender S, Singh K (2021). Environment, lifestyle, and female infertility. Reprod Sci.

[19] Lee C, Lee N (2019). Reproductive health promotion behavior of infertility women and normal women. Korean J Women Health Nurs.

[20] Guo Y, Liu Y, Yan X, Ding R, Tan H, Wang L (2022). Factors affecting the adoption of health-promoting behaviours in patients with polycystic ovary syndrome: a cross-sectional study. BMJ Open.

[21] Mirghafourvand M, Sehhati F, Rahimi M (2014). Health-promoting lifestyle and its demographic predictors in infertile couples referred to infertility clinic of Tabriz Al-Zahra Hospital, 2013. J Caring Sci.

[22] Kim M, Hong JE, Lee EY (2019). The relationship between fatigue, health-promoting behavior, and depression among infertile women. Korean J Women Health Nurs.

[23] Bektemur G, Keles E, Kaya L, Baydili KN (2024). Determinants of health-promoting behaviors in pregnant women. Rev Assoc Med Bras (1992).

[24] Jo J, Bang KS (2018). The effect of health promoting behavior on stress among resort workers. Korean J Occup Health Nurs.

[25] Mirghafourvand M, Charandabi SMA, Lak TB, Aliasghari F (2017). Relationship between health-promoting lifestyle and quality of life in women with polycystic ovarian syndrome. Int J Women Health Reprod Sci.

[26] Nagórska M, Lesińska-Sawicka M, Obrzut B, Ulman D, Darmochwał-Kolarz D, Zych B (2022). Health related behaviors and life satisfaction in patients undergoing infertility treatment. Int J Environ Res Public Health.

[27] Nho JH, Kim EJ (2022). Relationships among type-D personality, fatigue, and quality of life in infertile women. Asian Nurs Res (Korean Soc Nurs Sci).

[28] Kim YM, Nho JH (2020). Factors influencing infertility-related quality of life in infertile women. Korean J Women Health Nurs.

[29] Newton CR, Sherrard W, Glavac I (1999). The Fertility Problem Inventory: measuring perceived infertility-related stress. Fertil Steril.

[30] Kim JH, Shin HS (2013). A structural model for quality of life of infertile women. J Korean Acad Nurs.

[31] Hwang WJ, Hong OS, Rankin SH (2015). Predictors of health-promoting behavior associated with cardiovascular diseases among Korean blue-collar workers. Asia Pac J Public Health.

[32] Walker SN, Sechrist KR, Pender NJ (1995). Health promotion model – instruments to measure health promoting lifestyle: Health-Promoting Lifestyle Profile II (HPLP II) (adult version).

[33] Hayes AF (2018). Introduction to mediation, moderation, and conditional process analysis. A regression-based approach.

[34] Altiparmak S, Derya YA (2018). The effects of fertility-supporting health training on healthy lifestyle behaviors and infertility self-efficacy in infertile women: a quasi-experimental study. Eur J Integr Med.

[35] Swift A, Reis P, Swanson M (2021). Infertility stress, cortisol, coping, and quality of life in U.S. women who undergo infertility treatments. J Obstet Gynecol Neonatal Nurs.

[36] Yazdani F, Elyasi F, Peyvandi S, Moosazadeh M, Galekolaee KS, Kalantari F (2017). Counseling-supportive interventions to decrease infertile women’s perceived stress: a systematic review. Electron Physician.

[37] Soleimani R, Ansari F, Hamzehgardeshi Z, Elyasi F, Moosazadeh M, Yazdani F (2023). Perceived stress reduction through an infertility coaching program: a randomized controlled clinical trial. Sci Rep.

[38] Faramarzi M, Pasha H, Esmailzadeh S, Kheirkhah F, Heidary S, Afshar Z (2013). The effect of the cognitive behavioral therapy and pharmacotherapy on infertility stress: a randomized controlled trial. Int J Fertil Steril.

[39] Rooney KL, Domar AD (2018). The relationship between stress and infertility. Dialogues Clin Neurosci.

[40] Nery SF, Paiva SP, Vieira ÉL, Barbosa AB, Sant'Anna EM, Casalechi M (2019). Mindfulness-based program for stress reduction in infertile women: randomized controlled trial. Stress Health.

[41] Zaidouni A, Ouasmani F, Benbella A, Kasouati J, Bezad R (2019). The effect of nursing consultation based on Orem’s theory of self-care and Bandura’s concept on infertility stress. J Hum Reprod Sci.

[42] Song BK, Jee YJ (2021). Factors influencing fertility stress in infertile women. Asia-Pac J Converg Res Interchange.

[43] Kaya Y, Kizilkaya Beji N, Aydin Y, Hassa H (2016). The effect of health-promoting lifestyle education on the treatment of unexplained female infertility. Eur J Obstet Gynecol Reprod Biol.

[44] Wang W, Yang F, Bai Y, Lu Y, Mao X (2024). Association between domain-specific physical activity and mental health status after embryo transfer in IVF-ET-assisted pregnancy patients. Sci Rep.

[45] Khaled K, Tsofliou F, Hundley V, Helmreich R, Almilaji O (2020). Perceived stress and diet quality in women of reproductive age: a systematic review and meta-analysis. Nutr J.

[46] Malekpour P, Hasanzadeh R, Javedani Masroor M, Chaman R, Motaghi Z (2023). Effectiveness of a mixed lifestyle program in couples undergoing assisted reproductive technology: a study protocol. Reprod Health.

[47] Masoud OA, Madkour AMA, Abdelrhman MY, Zaghloul MG, Abd Elzaher OM, Abdelhafez AA (2023). Effect of maternity-led pro-fertility lifestyle intervention on health-promoting behaviors of women undergoing infertility treatment. Egyptian J Health Care.

[48] Lee S, Kim H (2017). Structural equation modeling on self-care behavior and quality of life in older adults with diabetes using citizen health promotion centers. J Korean Acad Nurs.

[49] McCarthy AL, Yates P, Shaban RZ (2013). Cross-sectional survey of the health behaviour of southeast Queensland women with cancer-treatment induced menopause: implications for cancer and primary care nurses. Collegian.

[50] Lee MK, Oh J (2020). Health-related quality of life in older adults: its association with health literacy, self-efficacy, social support, and health-promoting behavior. Healthcare (Basel).

[51] Seo EJ, Ahn JA, Hayman LL, Kim CJ (2018). The association between perceived stress and quality of life in university students: the parallel mediating role of depressive symptoms and health-promoting behaviors. Asian Nurs Res (Korean Soc Nurs Sci).

[52] Nho JH, Kim HY, Kim EJ (2022). Factors affecting quality of life in low-income overweight and obese women: the mediating effects of health-promoting behaviors. Worldviews Evid Based Nurs.

